# Calcitonin gene-related peptide monoclonal antibody treatment for new daily persistent headache

**DOI:** 10.1186/s10194-025-02111-2

**Published:** 2025-07-28

**Authors:** Sanjay Cheema, Khadija Rerhou Rantell, Rachel Pickering, Susie Lagrata, Salwa Kamourieh, Manjit Matharu

**Affiliations:** 1 Headache and Facial Pain Group, University College London (UCL) Queen Square Institute of Neurology, London, UK; 2 The National Hospital for Neurology and Neurosurgery, Queen Square, London, WC1N 3BG UK; 3 Education Unit, University College London (UCL) Queen Square Institute of Neurology, London, UK

**Keywords:** Chronic daily headache, CGRP, Migraine, New daily persistent headache

## Abstract

**Background:**

New daily persistent headache (NDPH) is a rare headache disorder that often resembles chronic migraine (CM) phenotypically, but unlike CM is daily from onset. Several calcitonin gene-related peptide monoclonal antibodies (CGRP mAbs) have been proven to be effective in CM. It is not known whether CGRP mAbs are effective in NDPH. We sought to assess the efficacy, tolerability, and safety of CGRP mAbs in NDPH and compare their effect in NDPH to CM.

**Methods:**

We performed an observational study using prospectively collected data in consecutive patients treated with CGRP mAbs in three groups: Group 1 included patients with NDPH with migraine features; Group 2 included patients with CM with daily headache; and Group 3 patients with non-daily CM. Given the observational nature of the study, propensity score matching was used in an attempt to balance the three groups on baseline factors to make them comparable. The majority of patients were treated with erenumab while the remainder received galcanezumab. Patients completed a headache diary and disability questionnaires at baseline and 12-week follow-up, with the primary endpoint being the proportion who achieved a reduction of at least 30% in monthly moderate-to-severe headache days (MSHD) compared between the three groups.

**Results:**

A total of 48 patients with NDPH, 101 with daily-CM, and 68 with non-daily-CM were included. From baseline to week 12, 11/47 (23%) of patients with NDPH had a ≥ 30% improvement in MSHD, compared to 46/99 (46%) in daily CM (OR 2.02, 95% CI 0.82–4.97, *p* = 0.125), and 51/61 (84%) in non-daily-CM (OR 4.41, 95% CI 1.17–16.6, *p* = 0.028). Only 5/47 (11%) of patients with NDPH had a ≥ 30% improvement in monthly headache days, and 24/44 (54%) reported an overall subjective improvement of ≥ 30%. Almost 50% of patients experienced at least one side effect, which were mild in almost all cases, and similar between groups.

**Conclusions:**

CGRP mAbs were effective in approximately 1/4 patients with treatment-refractory NDPH,but less likely to be effective in NDPH than CM. This suggests that NDPH cannot be seen as equivalent to CM and that new treatment options are required for this highly disabling disorder.

## Background

Calcitonin-gene related peptide (CGRP) is known to be involved in the pathophysiology of migraine and may also be relevant in other primary headache disorders. Monoclonal antibodies that block CGRP, or its receptor, have been proven effective in the preventive treatment of chronic migraine (CM) in several large randomised controlled trials [1–4]. CM is defined in the current version of the International Classification of Headache Disorders (ICHD-3) as headache on ≥ 15 days per month for > 3 months, of which ≥ 8 days per month have migraine features or respond to migraine-specific treatment [5].

New daily persistent headache (NDPH) is a headache syndrome that differs from CM in that it has a sudden onset and is continuous from the time of onset, often in people without a history of headache, and lasts at least three months [5]. NDPH is thought to be commonly refractory to migraine preventive treatments [6]. Despite the difference in onset, the symptoms of NDPH resemble CM in the majority of patients, and some have proposed that NDPH may represent a phenotypic variant of CM, though this remains a subject of ongoing debate [7].

If there is a significant overlap in the pathophysiology of NDPH and CM, then it may be expected that CGRP monoclonal antibodies would also be effective in patients with NDPH, or at least in the group who have migraine characteristics. Patients with NDPH have not been included in previous clinical trials of CGRP monoclonal antibodies, but some patients have anecdotally responded [8, 9]. Therefore, we sought to examine the response to CGRP monoclonal antibodies in NDPH in comparison to CM in a prospective study.

### Objectives

To assess the effectiveness and safety of anti-CGRP monoclonal antibody treatment in patients with NDPH in comparison to daily and non-daily CM.

## Methods

### Participants

We performed a prospective open-label study including consecutive patients with NDPH and CM who started treatment with CGRP monoclonal antibody treatment between January 2022 and March 2023. Patients were recruited from a single-centre secondary and tertiary headache clinic at the National Hospital for Neurology and Neurosurgery, London, UK.

Three groups of patients were included. Group 1 included patients with NDPH, who met ICHD-3 criteria [5] for NDPH, had phenotypic features of migraine, had an ongoing daily headache, had no symptoms to suggest a secondary cause for the headache, and had normal MRI brain imaging. Group 2 (daily-CM) included patients with chronic migraine, who had a daily headache. Group 3 (non-daily-CM) included patients with CM who had between 15 and 27 headache days per month. CM was divided into two groups so that there was a group (group 2) with comparable headache frequency and disability to the NDPH group at baseline.

As patients were treated under the UK National Health Service in accordance with National Institute for Health and Care Excellence (NICE) guidelines [10, 11], patients in all three groups were required to have failed to respond or unable to tolerate preventive medications in at least three classes of migraine preventive (including tricyclic antidepressants, beta blockers, topiramate, angiotensin receptor antagonists, sodium valproate, flunarizine, serotonin and norepinephrine reuptake inhibitors, pizotifen).

Patients were excluded if they had previously received a CGRP monoclonal antibody treatment prior to the study. No other changes to headache medication were made in the three months prior to treatment, or the first three months of CGRP treatment.

### Intervention

Most patients were treated with subcutaneous erenumab 140 mg once per month for three months. In light of postmarketing evidence suggesting a risk of hypertension with erenumab [12], all patients were required to measure their blood pressure prior to the treatment being prescribed, and patients with hypertension (defined as ≥ 140 systolic or ≥ 90 mmHg diastolic) were instead treated with galcanezumab 240 mg loading dose followed by 120 mg in one-month intervals for a further two doses.

### Data collection

Baseline information captured included demographics, onset of the headache disorder, comorbidities, and previous treatments that had been trialled.

A baseline headache diary was used to capture (over a four-week period): number of headache days, number of migraine days according to ICHD-3 criteria [5], mean headache severity (on a 0–10 verbal rating scale—VRS), moderate-to-severe headache days (defined as days with a VRS score of 4 or above), and number of days on which painkillers were taken.

Patients were also asked to complete the Headache Impact Test-6 (HIT-6) questionnaire of headache related disability [13], Hospital Anxiety and Depression Scale (HADS) [14], and the EQ-5D-5L quality of life questionnaire [15].

The same headache diary and questionnaires were completed for the 4-week period leading up to a 12 week follow up appointment. At the 12 week follow up patients were also asked to estimate their overall improvement on a scale of 0% (no improvement or worse) to 100% (complete resolution of migraine).

Patients were able to contact the clinical team with adverse events at any point in the study, and at the 12-week follow-up appointment patients were specifically asked to report any adverse effects.

### Outcome measures

The primary outcome measure was the proportion of patients with a ≥ 30% improvement in monthly moderate-to-severe headache days compared between the baseline four-week period to the four-week period preceding the 12-week follow-up date, compared between the three groups.

Planned secondary outcomes were improvement in headache days, migraine days, headache severity, headache load (a composite measure calculated as the monthly sum of headache VRS multiplied by headache hours for each day), HIT-6 score, EQ-5D-5L visual analogue scale (VAS), patient’s estimate of improvement, and frequency of adverse events.

Migraine days were later excluded from the analysis as it was suspected that some patients had not completed this section of the headache diary correctly, and therefore that this variable might not be reliable.

### Statistical analysis

All consecutive patients who met eligibility criteria during the period the study were included, and a sample size calculation was not performed. All data were collected prospectively within the clinical service using headache diaries and validated questionnaires. Missing data were not imputed.

Analyses were performed in R (R Studio version 4.3.3), IBM SPSS (Version 29.0), and Stata (Version 18).

Descriptive data were summarised using mean and standard deviation (SD) or median and interquartile range (IQR), depending on the distribution of data. Normality assumptions were assessed based on visual inspection of histograms.

For comparison of baseline data between the three groups we used Chi Squared (X^2^) test for categorical data, ANOVA (F) with Tukey post-hoc test for continuous data, and the equivalent non-parametric test, depending on the distribution of the data.

When analysing the outcome measures, comparisons between the three groups were made using the propensity score matching approach to control for imbalances between the three groups. Conditional on the propensity score, the distribution of pre-specified baseline covariates (gender, age, duration of chronic daily headache, number of failed preventive medications, and HAD-D depression score) will be similar between the three groups. Propensity score matching has been proven to be effective in reducing bias in observational studies where there are a number of potential confounders and dimensionality problems arise [16].

Propensity scores were estimated using generalized boosted models [17] in the twang package in R [18], which allows propensity score weighting to be implemented where there are multiple groups. Propensity score analysis used the average treatment effect (ATE), which was estimated using the “mnps” function, using absolute standardized mean difference as measure of balance. The ATE of a particular diagnostic group relative to an alternative diagnostic group is the comparison of the mean outcomes had the entire population been observed under the particular diagnostic group versus had the entire population been observed under the alternative diagnostic group. A combination of the overlap plot and the balance table were used to assess whether the groups were sufficiently similar.

Estimates of the differences of the outcome measures between the three groups were derived using negative binomial regression for analysis of monthly moderate-to-severe headache days and monthly headache days; linear regression for other continuous outcomes; and logistic regression for responder analysis; using the derived propensity score weights in the regression models.

For responder analysis, a greater than or equal to 30% response was used as the cut-off threshold to define responders for each of the outcomes based on headache diary metrics and patient estimate of improvement, and a greater than or equal to a 6-point improvement was used as the cut-off threshold for HIT-6 score.

### Ethical approval and consent

All patients gave informed consent for their anonymised data to be included in the research study. Research ethics committee approval was obtained from the London – Chelsea Research Ethics Committee, reference number 21/PR/0827.

## Results

### Participants

A total of 48 patients with NDPH and 169 patients with CM (of whom 101 had a daily headache) met inclusion criteria. Demographics and baseline headache diary results in the three groups are shown in Table 1.

**Table 1:** Baseline demographic and headache characteristics

	Group 1	Group 2	Group 3	Between groups comparison^a^
	NDPH *N* = 48	Daily-CM *N* = 101	Non-daily-CM *N* = 68	
Age in years, mean ± SD	46.4 ± 13.9	46.7 ± 13.3	49.9 ± 14.3	F = 1.38 *p* = 0.255
Females	31 (65%)	77 (77%)	57 (84%)	χ^2^ = 5.72 *p* = 0.057
Duration of CDH in years, median (IQR)	10.5 (10)	15 (12)	13 (16)	H = 2.56 *p* = 0.278
Preventives failed, median (IQR)	7.9 ± 2.9	6.8 ± 2.8	6.0 ± 2.2	H = 11.2 *p* = 0.004 (1 vs. 3)
Previous onabotulinumtoxinA	37 (77%)	72 (71%)	45 (66%)	χ^2^ = 16.6 *p* = 0.011
Headache days per month, median (IQR)	28 (0)	28 (0)	21 (9)	H = 199 *p* = < 0.001 (1 and 2 vs. 3)^#^
Moderate-to-severe headache days per month, median (IQR)	28 (1)	28 (6)	16 (8)	H = 89.0 *p* = < 0.001 (1 and 2 vs. 3)^#^
Headache severity (0–10 VRS), mean ± SD	7.0 ± 1.8	6.5 ± 1.7	4.9 ± 1.7	F = 24.6 *p* = < 0.001 (1 and 2 vs. 3)^#^
Monthly headache load, mean ± SD	3428 ± 1647	2759 ± 1474	1223 ± 1077	F = 40.0 *p* = < 0.001 (1 and 2 vs. 3)^#^
Monthly days abortives used, median (IQR)	2 (8)	7 (11)	9.5 (7)	H = 25.0 *p* = < 0.001 (1 and 2 vs. 3)^#^
Medication overuse (according to ICHD-3)	6 (13%)	32 (32%)	28 (41%)	χ^2^ = 11.1 *p* = 0.004 (1 vs. 3)
HIT-6 score, mean ± SD	65.4 ± 6.2	68.7 ± 5.3	66.8 ± 6.3	F = 5.84 *p* = 0.030
HADS-A score, mean ± SD	9.3 ± 5.1	11.1 ± 5.2	8.4 ± 5.4	F = 5.41 *p* = 0.005
HADS-D score, mean ± SD	9.2 ± 4.9	10.4 ± 5.1	8.0 ± 5.2	F = 4.41 *p* = 0.013
EQ-5D-5L-VAS score (0–100), mean ± SD	48.6 ± 17.7	40.8 ± 20.8	55.2 ± 22.2	F = 9.75 *p* = < 0.001 (2 vs. 3)
Pain catastrophising score (PCS), mean ± SD	23.6 ± 15.2	30.5 ± 14.0	26.0 ± 13.8	F = 4.31 *p* = 0.015
Drug received				χ^2^ = 13.87 *p* = 0.008
Erenumab	42 (88%)	63 (63%)	40 (59%)	
Galcanezumab	6 (12%)	38 (37%)	28 (41%)	

*Abbreviations*: *CDH* chronic daily headache, *CM* chronic migraine, *NDPH* new daily persistent headache, *IQR* interquartile range, *HADS* hospital anxiety and depression scale, *HIT-6* headache impact test-6, *NDPH* new daily persistent headache, *SD* standard deviation, *VRS* verbal rating scale

^a^Statistical tests presented are Chi Squared (χ^2^) for comparison of categorical data, ANOVA (F) with Tukey post-hoc test for comparison of parametric continuous data, and Kruksal-Wallis (H) with Dunn’s post-hoc test for comparison of non-parametric continuous data. No adjustment for multiple comparisons has been made

^#^On post-hoc testing these differences were only statistically significant between non-daily CM and both the other two groups and are expected as by definition the third group has fewer headache days, which all these variables are influenced by

Age distribution was similar between the three groups. As expected, there were fewer females in the NDPH group and medication overuse was less common. Headache diary metrics were similar in the NDPH and daily-CM groups, and lower in non-daily-CM, as expected, as they had fewer headache days by definition. Disability, quality of life, anxiety, and depression scores all appeared worst in the daily-CM group and best in the non-daily CM at baseline, with the NDPH group having intermediate scores. There was a higher proportion of patients with NDPH prescribed erenumab than in both the CM groups (see Table 1).

A small proportion of patients (one in the NDPH group, two in the daily-CM group, and seven in the non-daily CM group) did not return the 12-week interval diaries, and six NDPH patients, eight daily-CM patients, and nine non-daily CM patients did not return the disability questionnaires.

### Effectiveness

**Fig. 1:**
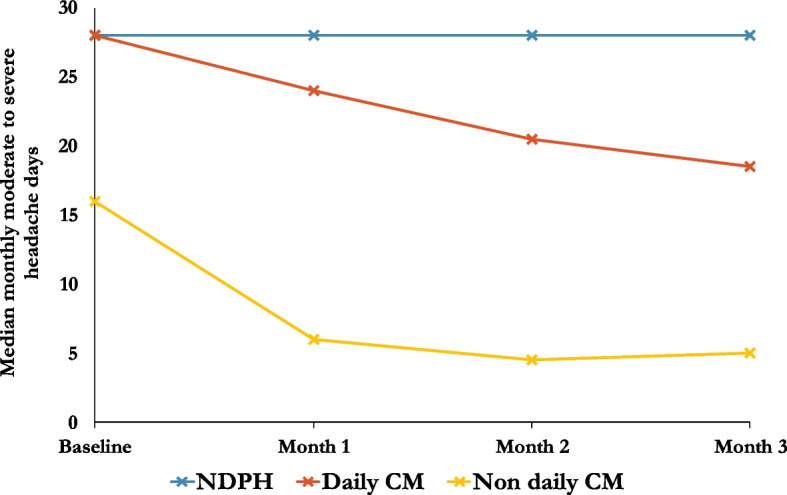
Improvement in moderate-to-severe headache days over first 3 months of treatment

In the NDPH group, 11/47 (23%) of patients had a ≥ 30% improvement in monthly moderate-to-severe headache days. This compared to 46/99 (46%) in daily CM (OR 2.02, 95% CI 0.82–4.97, *p* = 0.125), and 51/61 (84%) in non-daily-CM (OR 4.41, 95% CI 1.17–16.6, *p* = 0.028). Figure 1 shows the response rates over the first three months of treatment.

**Fig. 2:**
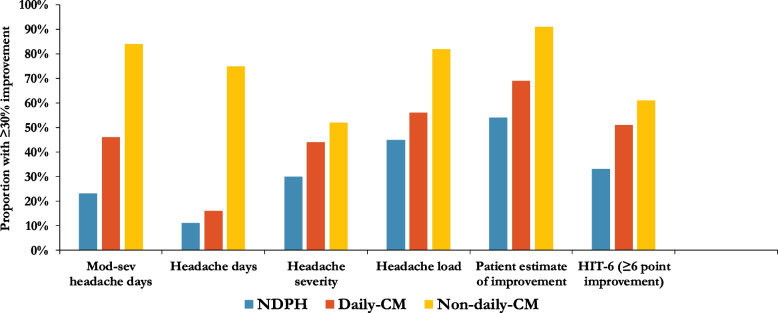
Proportion of responders for each outcome measure

Improvement in headache days was rare in both NDPH (5/47, 11%) and daily CM (16/99, 16%), compared to 46/61 (75%) in non-daily CM (OR 7.44, 95% CI 1.47–37.8, *p* = 0.015). There was no difference in headache severity between the groups. Results of all primary and secondary outcomes are summarised in Table 2 and Fig. 2.

**Table 2:** Outcome measures at 12 week follow up and between group comparisons adjusted by propensity score

	Group 1	Group 2	Group 3
	NDPH *N* = 48	Daily-CM *N* = 101	Non-daily-CM *N* = 68
Moderate-to-severe days (0–28)			
Median change (IQR)	0 days (7)	−5 days (13)	−9 days (9)
Mean change (SD)	−5.3 days (9.5)	−7.1 days (8.3)	−9.6 days (6.6)
Regression co-efficient (95% confidence interval)	Comparison group (CG)	−1.19 (−4.71, 2.33)	−4.94 (−9.63, −0.25)
*P* value	CG	0.505	0.039
Patients with ≥ 30% improvement	11/47 (23%)	46/99 (46%)	51/61 (84%)
Odds ratio (95% confidence interval)	CG	2.02 (0.82, 4.97)	4.41 (1.17, 16.6)
*P* value	CG	0.125	0.028
Headache days (0–28)			
Median change (IQR)	0 days (0)	0 days (2)	−10 days (10)
Mean change (SD)	−2.2 days (6.3)	−3.8 days (8.2)	−6.8 (11.2)
Regression co-efficient (95% confidence interval)	CG	−1.19 (−3.72, 1.34)	−9.38 (−14.5, −4.26)
*P* value	CG	0.204	< 0.001
Patients with ≥ 30% improvement	5/47 (11%)	16/99 (16%)	46/61 (75%)
Odds ratio (95% confidence interval)	CG	1.46 (0.44, 4.88)	7.44 (1.47, 37.8)
*P* value	CG	0.535	0.015
Severity (0–10 VRS)			
Mean change (SD)	−1.4 (1.9)	−1.8 (2.0)	−1.6 (2.5)
Regression co-efficient (95% confidence interval)	CG	−0.63 (−1.29, 0.02)	−0.86 (−1.82, 0.09)
*P* value	CG	0.059	0.076
Patients with ≥ 30% improvement	14/47 (30%)	44/99 (44%)	32/61 (52%)
Odds ratio (95% confidence interval)	CG	1.61 (0.72, 5.60)	1.69 (0.63, 4.54)
*P* value	CG	0.248	0.295
Headache load			
Mean change (SD)	−783 (1173)	−953 (1197)	−770 (841)
Regression co-efficient (95% confidence interval)	CG	−404 (−818, 11.4)	−682 (−1205, −159)
*P* value	CG	0.057	0.011
Patients with ≥ 30% improvement	21/47 (45%)	55/99 (56%)	50/61 (82%)
Odds ratio (95% confidence interval)	CG	1.36 (0.65, 2.85)	3.54 (1.27, 9.89)
*P* value	CG	0.419	0.016
Patient estimate of improvement (0–100)			
Mean change (SD)	34.0 (28.8)	40.7 (29.5)	64.8 (26.8)
Regression co-efficient (95% confidence interval)	CG	6.20 (−5.14, 17.54)	27.6 (16.23, 38.93)
*P* value	CG	0.282	< 0.001
Patients with ≥ 30% improvement	24/44 (54%)	66/95 (69%)	53/58 (91%)
Odds ratio (95% confidence interval)	CG	1.83 (0.83, 4.03)	10.8 (3.47, 33.8)
*P* value	CG	0.135	< 0.001
HIT-6 score			
Mean change (SD)	−4.6 points (8.3)	−8.2 points (13.7)	−8.0 points (14.3)
Regression co-efficient (95% confidence interval)	CG	0.59 (−2.72, 3.89)	−2.03 (−5.67, 1.62)
*P* value	CG	0.727	0.273
Patients with ≥ 6 point improvement	14/42 (33%)	47/93 (51%)	36/59 (61%)
Odds ratio (95% confidence interval)	CG	1.19 (0.47, 2.97)	2.69 (1.00, 7.25)
*P* value	CG	0.729	0.051
EQ-5D-5L VAS			
Mean change (SD)	3.5 points (22.4)	7.7 points (21.6)	12.6 points (20.9)
Regression co-efficient (95% confidence interval)	CG	1.62 (−6.59, 9.84)	9.24 (0.06, 18.4)
*P* value	CG	0.697	0.048

*Abbreviations*: *CM* chronic migraine, *NDPH* new daily persistent headache, *IQR* interquartile range, *HIT-6* headache impact test-6, *NDPH* new daily persistent headache, *SD* standard deviation, *VRS* verbal rating scale

Patient estimate of improvement differed between the three groups, in line with the difference in the headache diary metrics, with the NDPH and daily-CM groups experiencing a mean improvement of 34% and 41% respectively, whereas the non-daily CM reported a mean improvement of 65% (coefficient = 6.95, 95% CI 3.52–10.38, *p* < 0.001). Patient estimate of improvement was strongly correlated with improvement in moderate-to-severe headache days (*R* = −0.670, *p* < 0.001).

Due to the imbalance in the proportion of patients who received each drug, a secondary analysis was performed comparing response rates between erenumab and galcanezumab, across all the patients included in the study. This showed similar response rates (50% and 45% improvement in moderate-to-severe headache days respectively, χ^2^ = 1.78, *p* = 0.411), suggesting that this did not significantly influence the results.

### Adverse events

Approximately half of patients experienced at least one adverse effect. The most common adverse event in all three groups was constipation, affecting almost a quarter of patients overall (see Table 3). One patient with daily-CM had a severe allergic rash, and one patient with NDPH had a severe flare of inflammatory arthritis soon after starting erenumab. In both patients, CGRP treatment was stopped and the adverse effect improved with appropriate treatment and cessation of CGRP monoclonal antibody therapy. There were no other serious adverse events.

**Table 3:** Adverse events

	NDPH *n* = 48	Daily-CM *n* = 101	Non-daily-CM *n* = 68	Total *n* = 217
Constipation	7 (15%)	26 (26%)	18 (26%)	51 (24%)
Fatigue	2 (4%)	19 (19%)	10 (15%)	31 (14%)
Cold/flu-like symptoms	4 (8%)	11 (11%)	7 (10%)	22 (10%)
Hair loss	3 (6%)	10 (10%)	5 (7%)	18 (8%)
Injection site reaction	1 (2%)	9 (9%)	5 (7%)	15 (7%)
Nausea	3 (6%)	6 (6%)	2 (3%)	11 (5%)
Pruritis	0	6 (6%)	4 (6%)	10 (5%)
Muscle spasms	1 (2%)	3 (3%)	1 (1%)	5 (2%)
Rash	0	2 (2%)	2 (3%)	4 (2%)
Hypertension	1 (2%)	2 (2%)	1 (1%)	4 (2%)
Raynaud’s	1 (2%)	0	0	1 (0%)
Other	Back pain Dizziness Flare of inflammatory arthritis Palpitations	Diarrhoea Dizziness × 2 Facial twitching Vivid dreams Weight gain × 2 Worsening of PoTS symptoms	Abdominal pain Bloating Breathlessness Insomnia Joint pain × 3 Low mood × 2 Mouth ulcers Neck stiffness Sore throat Tremor Weight gain	
None	28 (58%)	49 (49%)	31 (46%)	108 (50%)

## Discussion

This study has shown that anti-CGRP monoclonal antibodies have little effect on headache days, but improvement in moderate-to-severe headache days in approximately one in four patients and a subjective improvement of symptoms in approximately half of patients with NDPH. The probability of improvement in the primary outcome measure of moderate-to-severe headache days in NDPH was approximately half of that in daily CM and a quarter of that in non-daily CM.

This contrasts with their efficacy in other chronic daily headache syndromes. Anti-CGRP monoclonal antibodies are proven to be effective for CM in several randomised controlled trials [1–4], and better tolerated than oral preventive medications such as topiramate [19]. Observational studies have also shown anti-CGRP monoclonal antibodies are effective in a proportion of patients with persistent post-traumatic headache and persistent headache associated with idiopathic intracranial hypertension [20, 21]. However, this study included a highly treatment-refractory group which had failed several preventive treatments, and the majority had failed treatment with onabotulinumtoxinA, meaning that the response cannot necessarily be attributed to placebo, and may still be clinically useful in the absence of other available treatment options.

We also showed that these treatments are less effective in patients with CM who have a daily headache than those who have between 15–27 headache days per month. This corroborates a previous study that showed that response was 56% in daily-CM and 90% in non-daily CM [22].

No previous study has evaluated the efficacy of CGRP monoclonal antibody treatment specifically for NDPH. A retrospective study including 112 adolescents with a mixed population of chronic headache disorders, who were given either erenumab, fremanezumab, or galcanezumab, included 12 patients with NDPH [23]. There was a significant benefit in approximately 33/112 (29.5%) of the total population, but response rate in the NDPH group was not reported independently. Another retrospective study reported response to erenumab in 82 patients with “abrupt onset continuous and unremitting daily headache disorders”, of whom 53 had NDPH [24]. Standardised outcome measures were not used, but the authors found that 34% of patients with NDPH reported a subjective improvement in severity of headache and migraine symptoms following treatment. In comparison, the current study was prospective, included clearly defined patient groups, and used standardised outcome measures as recommended by IHS guidelines [25].

The results of this study showing that CGRP monoclonal antibodies are less likely to be effective in NDPH than CM, suggest that it is possible that CGRP plays less a role in the pathophysiology of primary NDPH than in CM. CGRP is a neuropeptide which is widely expressed throughout the nervous system. Its role in migraine is believed to involve release following activation of trigeminovascular fibres innervating the dura, contributing to the initiation of migraine attacks [26]. It may therefore be less relevant in headache which is daily and continuous (whether NDPH or daily-CM).

The difference in treatment response in this study contrast with a recent study evaluating onabotulinumtoxinA treatment for NDPH compared to daily and non-daily CM using similar methodology where there was no significant difference in headache diary metrics or patient reported improvement between NDPH and either of the CM groups [27]. This could be explained by the mechanism of action of onabotulinumtoxinA and CGRP monoclonal antibody treatment in these disorders. OnabotulinumtoxinA inhibits soluble N-ethylmaleimide-sensitive fusion attachment protein receptor (SNARE) proteins in motor and sensory neuron synapses, thereby reducing exocytosis of several neurotransmitters and proteins. It has also been shown to reduce the number of pain-sensitive ion channels such as transient receptor potential cation channel subfamily V member 1 (TRPV1) in neurons [28]. Its efficacy in chronic migraine is therefore hypothesised to be due to this reduction in both activation of pain sensing neurons via the reduction in transmitting neuropeptides and the downregulation of ion channels thought to be involved in sensitisation to pain. CGRP monoclonal antibodies block just one of the neuropeptides involved in pain transmission in trigeminal neurons i.e. CGRP. The transmission of pain signals in trigeminal neurons is conducted via both thinly myelinated A-delta-fibres, which transmit short-lasting acute pain signals; and unmyelinated C-fibres, which transmit longer-lasting duller pain signals. OnabotulinumtoxinA has been shown to selectively inhibit C-fibres, but not A-delta-fibres, in pre-clinical models [29]. Similarly, pre-clinical studies suggests that CGRP monoclonal antibodies may preferentially affect A-delta-fibres, although this has not been confirmed in humans [30]. Patients with NDPH generally do not experience acute attacks of pain and acute analgesics are rarely helpful [31]. This difference in mechanism of action may help to explain why CGRP monoclonal antibodies were very effective in non-daily CM, but not in NDPH, and also why onabotulinumtoxinA appears to be more effective than CGRP monoclonal antibodies in NDPH. A new class of small molecule CGRP receptor antagonists (gepants) is now available for migraine. No published data is yet available on their efficacy in NDPH with migraine features. Knowledge of whether they are effective or not in NDPH would future increase knowledge on the mechanisms and treatment of NDPH.

We showed a high rate of mild adverse effects of CGRP monoclonal antibody treatment, although this is comparable to the overall adverse event rate seen in trials of erenumab and galcanezumab in CM [2, 3, 32]. Constipation and fatigue were more common in our study than in the original trials, but this aligns with other real-world experience studies of erenumab for CM [33, 34]. The rate of adverse effects with CGRP monoclonal antibodies was higher than the rate found for onabotulinumtoxinA in a similar real-world study in a similar population [27]. However, as these findings are based on separate observational data, direct comparisons should be interpreted cautiously. Where both treatments are available, onabotulinumtoxinA may be considered earlier in the treatment pathway due to its favourable tolerability profile.

Despite limited changes in headache frequency among patients with NDPH, over half reported subjective improvement following treatment with CGRP monoclonal antibodies, and one-third experienced clinically meaningful improvements in disability scores. This discrepancy between objective diary metrics and perceived benefit warrants closer scrutiny. Our findings raise the possibility that CGRP-targeted therapies may confer benefit in NDPH through mechanisms beyond simple attack reduction. Notably, approximately 30 percent of patients experienced a 30 percent or greater reduction in headache severity, suggesting that improvement in pain intensity may contribute meaningfully to perceived clinical benefit. Additionally, symptoms such as nausea, vomiting, fatigue, cognitive fog, and sensory hypersensitivity, which are frequently reported in NDPH, may also respond to CGRP blockade. Unfortunately, monthly migraine days could not be included in the analysis as intended, and may have reflected this change in non-headache symptoms. Migraine days and non-headache symptoms would be helpful for any future studies to include as outcome measures.

The psychological impact of initiating a novel, biologically targeted therapy, particularly in patients with long-standing, treatment-refractory headache, may further influence patient-reported outcomes. The experience of receiving a modern, mechanism-based intervention can foster hope, enhance engagement, and restore a sense of control over the condition, which may in turn amplify perceptions of improvement even in the absence of major objective changes. These observations highlight the importance of including structured evaluation of non-headache symptom domains in future studies of NDPH, to better understand the nature of treatment response and to inform more comprehensive outcome measures.The inability to control for bias is a limitation of the study design and the use of propensity score was an attempt to mimic some of the characteristics of a randomised study. Randomisation avoids systematic differences between groups with respect to known or unknown baseline variables that could affect outcome. As this study was not a randomised controlled trial, there were some differences in baseline characteristics between the groups. As in the previous onabotulinumtoxinA study [27], we accounted for the largest confounder by separating patients with CM into those who did and did not have a daily headache, so that the daily-CM group would be more easily comparable to the NDPH group. However, some differences in baseline characteristics existed due to known differences between NDPH and CM, such as the greater female predominance in CM than NDPH [31], and whilst we accounted for these differences using propensity score matching, other unknown confounders may have existed.

The absence of a control arm in this study makes it impossible to discriminate between outcomes that are the consequence of a treatment from those caused by other factors. In particular, the absence of of a placebo group limiting limits absolute conclusions about the true magnitude of treatment effect i.e. about whether there is a mild effect of CGRP monoclonal antibodies in NDPH from being made. The duration of follow up of 12 weeks was chosen due to the NICE criteria under which patients were treated which state that treatment should be stopped after 12 weeks if there is < 30% improvement in headache frequency. It is known that some patients can take > 24 weeks to respond to CGRP monoclonal antibody treatments [35], and such patients would not have been detected with the current study. Furthermore, there is also the issue of generalisability of the study results as data came from a single centre which may not be fully representative of the target population. The current study was not powered for hypothesis testing, and the patient numbers limiting power to therefore the study may lack power to detect small differences in treatment response. Despite NDPH is being a rare disorder, with the best estimate of its prevalence being 0.03% of the general population [36], a multicentre, randomised, controlled study should be considered in the future.

## Conclusions

CGRP monoclonal antibody treatment appears to be less effective in NDPH than CM, particularly patients with CM who do not have a daily headache. This suggests that NDPH should not be regarded as equivalent to CM and that new treatment options are required for treatment-refractory NDPH. However, given the frequently refractory nature of NDPH, a response rate of 23% and mean patient perceived improvement of 34% may still be considered clinically significant. Given the limitations inherent to the study design, the effect of CGRP monoclonal antibody treatment in NDPH should be formally evaluated in randomised controlled studies. A greater understanding of the pathophysiological basis of NDPH and its relationship to other primary headache disorders would aid the development of new treatment approaches.

## Data Availability

Anonymised data used in this study are available from the corresponding author upon reasonable request by any qualified investigator.

## Abbreviations

ANOVA: Analysis of variance
ATE: Average treatment effect
CGRP: Calcitonin gene-related peptide
CM: Chronic migraine
EQ-5D-5L: EuroQol-5 Dimensions-5 Levels
HADS: Hospital anxiety and depression scale
HIT-6: Headache impact test 6
MSHD: Moderate-to-severe headache days
NDPH: New daily persistent headache
SD: Standard deviation
VRS: Verbal rating scale
